# Detection and tracking of ocean layers using an AUV with UKF based extremum seeking control in the Baltic Sea

**DOI:** 10.1038/s41598-024-70775-y

**Published:** 2024-09-04

**Authors:** Tim Benedikt von See, Jens Greinert, Thomas Meurer

**Affiliations:** 1 Deep Sea Monitoring Group, GEOMAR Helmholtz Centre for Ocean Research Kiel, 24148 Kiel, Germany; 2 Institute of Geosciences, Kiel University, 24118 Kiel, Germany; 3 Digital Process Engineering Group, Institute for Mechanical Process Engineering and Mechanics, Karlsruhe Institute of Technology, 76187 Karlsruhe, Germany

**Keywords:** Physical oceanography, Engineering, Scientific data, Computational science

## Abstract

Adaptive sampling and situational awareness are key features of modern autonomous underwater vehicles (AUVs) since data quality can be improved while operation time and cost can be reduced. An example for adaptive sampling in the marine environmental context is thermocline detection and tracking. The thermocline as horizontal ocean layer separates warm and cold water and is a key feature in many marine disciplines. For example, it influences the distribution and exchange of nutrients and is a habitat for many organisms. In this paper we use an unscented Kalman Filter (UKF) based extremum seeking control (ESC) to find and follow ocean layers such as the thermocline. Computer simulations and real-world tests show that the method is able to find and track non-trivial real-world ocean layers with sensors subject to hysteresis and delay effects.

## Introduction

Ocean layers can be divided into mainly vertical and mainly horizontal zones where horizontal layers are generally much thinner than vertical ones. Examples for the latter are upwelling fronts or the sides of ocean eddies and examples for horizontal ocean layers are the thermocline, halocline, oxycline, and pycnocline. These two layer types occur due to different natural phenomena but in principle they are all layers that separate two water layers of different water properties and can thus be characterized by a distinct gradient of the particular water property with respect to the vertical or horizontal distance. Such layers are key features of many marine disciplines. Upwelling fronts for example transport cold and often nutrient rich bottom water to the surface and thereby lead to increased primary productivity making these regions important fishing grounds^1^. Eddies often form in coastal regions and detach from the coast transporting nutrient-rich water into the open ocean and are believed to play an important role in the $\text {CO}_2$ uptake of the ocean^2,3^. Horizontal ocean layers on the contrary do not indicate transport of water but in most cases rather minimize the exchange between two water layers. The pycnocline, e.g., is the layer that separates two water layers of different densities and can be a barrier for sinking particles thus slowing down the nutrient transport towards the seafloor. Similarly the thermocline, halocline, and oxycline are the layers that separate water layers of different temperatures, degree of salinity and oxygen content, respectively. In the following, horizontal ocean layers are the main focus, nevertheless the method proposed in this paper is also applicable to vertical ocean layers.

The first papers that dealt with autonomous detection and tracking of horizontal ocean layers targeted the thermocline and aimed at shortening the classical yo-yo trajectory between the surface and seafloor. Petillo et al. (2010) propose a method, where first a complete dive of the water column is performed to calculate the average gradient of the temperature with respect to depth. The thermocline is defined as the water layer, where the temperature gradient is larger than the average gradient. A yo-yo trajectory is planned in this layer and a restart with a complete dive is performed at half the characteristic time of the feature to account for large scale variations of the thermocline, e.g., due to heating of the water by solar radiation during the day^4^. The choice of the average gradient as characterization of the thermocline can be suboptimal in complex scenarios. If two layers are present, divided by a well mixed layer, the method will only cover one of them. In Cruz et al. (2010a) a state machine consisting of four states is used to track the thermocline based on the temperature gradient. The upper and lower limit of the thermocline are detected via pre-defined thresholds of the gradient that are updated based on the maximum gradient found on the last ascent or descent leg, respectively. The method is applied to real conductivity, temperature and depth (CTD) data in a simulation environment^5^ and in Cruz et al. (2010b) field data from a demonstration in a dam reservoir is presented^6^. Due to the dynamic update of the thresholds this method leads to significant changes in the sampled layer depth and is prone to over- or underestimate the layer thickness. Zhang et al. (2010) propose a peak gradient method for thermocline detection, where they divide the water column into depth bins and calculate the temperature gradient of neighboring bins. The mean thermocline depth is defined to be at the largest gradient. A yo-yo trajectory is planned in this depth plus/minus an extension depth to make sure that the whole thermocline is covered^7^. Simulation results based on real CTD data are presented followed by data from AUV dives in Monterey Bay, CA in Zhang et al. (2012)^8^. This method yields a sampled region that is symmetric around the maximum gradient, which in nature often does not represent the actual ocean layer, especially not in the case of complex water layering. Feng et al.^9^ propose a threshold-based method that is more conservative than the ones mentioned so far. Here, the threshold for the temperature gradient that defines the thermocline is set in advance by the operator. A complete dive is performed and the minimum and maximum depth values at which gradients above the threshold were measured are saved. The next ascend or descend phase are planed between these depth values plus/minus an extension depth^9^. Thereby more than one boundary layer can be sampled, regardless of the distance between them. The proposed method is compared to the approaches of Petillo et al. (2010) and Zhang et al. (2012) in a simulation and results from field tests are presented. This approach relies heavily on the correct a priori estimation of the temperature gradient threshold as it is not updated or calculated dynamically during the AUV dive as in the approaches above.

The described approaches are similar in that they use direct thresholding of the temperature gradient with respect to depth. An approach that differs from this is presented in Antunes and Cruz (2019). The authors propose to use an ESC loop based on Krstić and Wang (2000)^10^ in combination with a vertical profiler^11^. Extremum seeking control is a signal based optimization technique that does not require a proper mathematical model of the process under consideration. The basic idea of ESC is to add a periodic perturbation signal to the system input, which leads to changes of the measurable system output that are mapped to a properly chosen cost function. The gradient of the cost function with respect to the system input is estimated and used to drive the system to the working point corresponding to either the maximum or the minimum of the cost/objective function. In Antunes and Cruz (2019) the temperature gradient is used as the cost function for the ESC and it is calculated based on a vertical sensor array with two sensors. The approach is applied to two artificial functions and to real temperature data in a simulation environment^11^. However, this data is very smooth, hence a distinct gradient is present in nearly all working points, which is often not the case in nature.

The approach presented in this paper is based on our earlier work von See et al. (2021) which also uses an ESC scheme for thermocline tracking. Here, an UKF is used as gradient estimator in the ESC loop, which has the advantage that the convergence speed does not depend on the amplitude of the perturbation signal as it is the case in other commonly used ESC approaches^12^. Furthermore measures are taken to deal with situations, where no gradient is measurable. The contribution of this paper lies in a number of improvements over our previous work and the validation of the approach through a field trial. The improvements are that a full dive of the AUV is added at the beginning to normalize the cost function, thereby reducing the need for situation dependent tuning, and that a state machine is introduced to reset the UKF based ESC in case of a sudden change of the layer parameters or if the UKF based ESC has driven the AUV into a local maximum or minimum, thereby increasing its robustness and reliability. In addition, the approach is applied to more realistic sensor data. This is achieved by analysing the sensor effects of a real CTD, which are similar to magnetic hysteresis. The analysis shows that a single temperature-depth profile as used in von See et al. (2021) is not sufficient for a realistic sensor simulation. Consequently, the sensor effects are emulated in the simulation environment in this paper. Furthermore, in the raw data used here, there are two ocean layers spanning a larger depth range. This shows that the method is capable of detecting and tracking more than one ocean layer, even in the presence of sensor delays. Finally, results from a field test with an AUV in the Baltic Sea in the presence of complex water layering are presented, which validate the proposed method.

The paper is structured as follows. In the “Methods” section the methodology is shortly explained and adapted to the use case of thermocline tracking followed by the AUV dynamics considered in the simulation as well as the simulation framework, simulation data and sensor effect emulation. The results of the simulation and the field test are presented in the “Results” section followed by their evaluation and comparison with related methods in the “Discussion” section.

## Methods

### Extremum seeking control

**Fig. 1:**
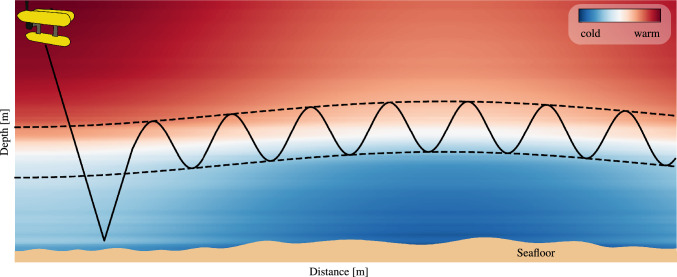
Illustration of adaptive sampling with an AUV in the case of thermocline tracking with variations in the boundary layer. The depth is plotted against the distance with color coded temperature. The black dashed lines indicate the region of scientific interest that shall be sampled and the solid black line shows the desired AUV path.

To illustrate the operation of the approach a sketch of the autonomous detection and tracking of the thermocline is shown in Fig. 1. Here the depth is plotted against the distance with color coded temperature. The boundary layer which separates the warmer surface water and the cooler bottom water slightly changes with the distance. The region that shall be sampled is bounded by the black dashed lines and the desired AUV path is plotted as the black solid line.

**Fig. 2:**
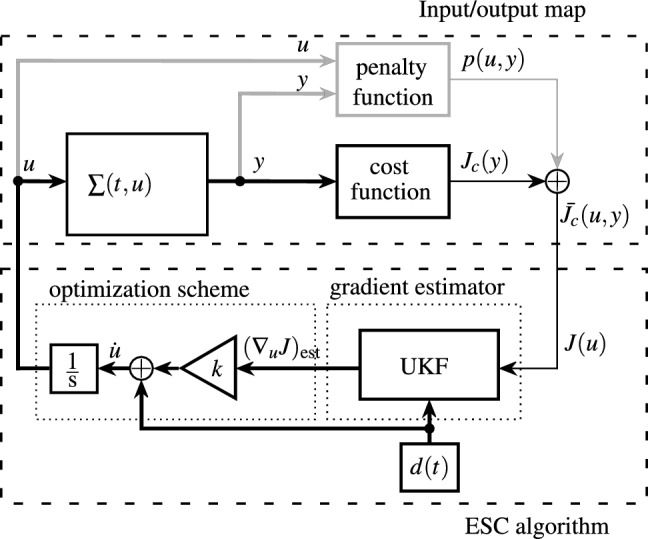
ESC loop for an asymptotically stable system $\Sigma (t,\varvec{u})$ with input $\varvec{u}$, output $\varvec{y}$, cost function $\bar{J}(\varvec{u},\varvec{y}) = J(\varvec{u})$ and penalty function in gray, proposed in Lutz et al. (2019)^12^.

The method that is used for this study is the ESC loop shown in Fig. 2, which is based on Lutz et al. (2019)^12^. It is build up of an input/output map at the top and the ESC algorithm at the bottom. The first consists of the unknown nonlinear time variant system $\Sigma (t,\varvec{u}):\mathbb {R}^+_0 \times \mathbb {R}^m \rightarrow \mathbb {R}^p$ with the system input vector $\varvec{u}(t) \in \mathbb {R}^m$ and the measurement vector $\varvec{y}(t) \in \mathbb {R}^p$, the cost function $J_c(\varvec{y})$, and the penalty function $p(\varvec{u,y})$, which is integrated to handle constraints. Two assumptions about the system $\Sigma (t,\varvec{u})$ have to be taken into account:

#### Assumption 1

The system is either asymptotically stable or stabilized by an underlying control loop.

#### Assumption 2

The time scales of the system dynamics and the forcing signal are separable so that the influence of the system dynamics on the cost function can be neglected.

A requirement for the application of ESC is that the cost function depends on the system input. By *Assumption 2* the relation $J_c(\varvec{y}) + p(\varvec{u,y}) = \bar{J}(\varvec{u},\varvec{y}) = J(\varvec{u})$ must hold true at least on a small time scale.

The cost function $J(\varvec{u})$ and the perturbation signal $\varvec{d}(t)$ serve as input for the gradient estimator, here an UKF. The output of the UKF is the estimated gradient of the cost function with respect to the system input in the current point of operation $(\nabla _{\varvec{u}} J)_\text {est}$, which is multiplied by the gain *k* and added to the perturbation signal. The sum is integrated and used as the new desired system input to drive the system to the working point corresponding to the maximum or minimum of the cost function for $k>0$ and $k<0$, respectively. To make this work self-contained and to motivate the application, the approach proposed in Lutz et al. (2019)^12^ is briefly recalled and summarized. Starting with the cost function $J(\varvec{u})$ its rate of change can be determined using the chain rule, which yields

1 $$\begin{aligned} \frac{\text {d} J(\varvec{u})}{\text {d}t} =\left( \frac{\text {d}\varvec{u}}{\text {d}t}\right) ^\text {T} \nabla _{\varvec{u}} J(\varvec{u}) = [\dot{u}_1 \cdots \dot{u}_m] \begin{bmatrix} \frac{\partial }{\partial u_1} J(\varvec{u})\\ \vdots \\ \frac{\partial }{\partial u_m} J(\varvec{u}) \end{bmatrix}, \end{aligned}$$

where $\nabla _{\varvec{u}} J(\varvec{u})$ denotes the gradient of the cost function with respect to the input vector. This vector of partial derivatives of the cost function with respect to the $m=n-1$ components $u_i$ of the input vector appended by the cost function is chosen as the states $\varvec{x}(t) \in \mathbb {R}^n$ of the UKF estimator, thus

2 $$\begin{aligned} \varvec{x} = \begin{bmatrix} \frac{\partial }{\partial u_1} J(\varvec{u})\\ \vdots \\ \frac{\partial }{\partial u_m} J(\varvec{u})\\ J(\varvec{u}) \end{bmatrix} = \begin{bmatrix} x_1\\ \vdots \\ x_{n-1} \\ x_n \end{bmatrix}. \end{aligned}$$

In most cases the time variant system $\Sigma (t,\varvec{u})$ is unknown apriori, therefore the gradient’s time derivative $\dot{\varvec{x}}$ is modeled as additive white process noise $\hat{\varvec{w}} = [w_1, \ldots , w_{m}]^\text {T}$ with covariance $Q \in \mathbb {R}_+^{m \times m}$. Accordingly the estimator model

3 $$\begin{aligned} {\dot{{\varvec{x}}}} = \begin{bmatrix} 0\\ \vdots \\ 0\\ {\dot{{\varvec{u}}}}^\text {T}H\varvec{x} \end{bmatrix} + \begin{bmatrix} w_1\\ \vdots \\ w_{m}\\ 0 \end{bmatrix}, \quad t > t_0, ~ \varvec{x}(t_0) = \varvec{x}_0 \end{aligned}$$

with $H = [I_{m},\varvec{0}_{m}] \in \mathbb {R}^{m \times n}$ is obtained, where $I_{m} \in \mathbb {R}^{m \times m}$ is the identity matrix and $\varvec{0}_{m}$ is the *m*-dimensional zero vector. The time derivative of the system input $\dot{\varvec{u}}(t) \in \mathbb {R}^{m}$ is obtained by integrating (1) and (2) into the ESC algorithm according to Fig. 2 and reads

4 $$\begin{aligned} \dot{\varvec{u}} = \varvec{d} + k(\nabla _{\varvec{u}} J)_\text {est} = \varvec{d} + k H \varvec{x}. \end{aligned}$$

Here $\varvec{d}(t) \in \mathbb {R}^{m}$ denotes a periodic perturbation signal. Integrating (4) into (3) with $\varvec{w} = [\hat{\varvec{w}}^\text {T}\; 0]^\text {T}$ leads to the full process model of the gradient estimator

5a $$\begin{aligned} \begin{aligned} \dot{\varvec{x}}&= \begin{bmatrix} 0\\ \vdots \\ 0\\ \varvec{d}^\text {T} H \varvec{x} + k \varvec{x}^\text {T} H^\text {T} H \varvec{x} \end{bmatrix} + \varvec{w} =\varvec{f}(\varvec{x}, \varvec{d}) + \varvec{w}, \quad t > t_0, \quad \varvec{x}(t_0) = \varvec{x}_0. \end{aligned} \end{aligned}$$

The measurement equation of the system $\Sigma (t,\varvec{u})$ is given by

5b $$\begin{aligned} y&= J(\varvec{u}) + l, \end{aligned}$$

with *l* denoting white measurement noise with covariance $R > 0$. Consequently the output equation of the estimator is chosen as

5c $$\begin{aligned} \hat{y}&= \hat{x}_n + l = h(\hat{\varvec{x}}) + l. \end{aligned}$$

For state estimation given the nonlinear process model (5) a nonlinear filter is set up and integrated into the approach. The UKF is used here because it is easier to implement and promises more accurate capturing of the nonlinearities^13^. The working principle and a discussion of the UKFs properties is given in the Supplementary material.

### Application of the UKF based ESC for ocean layer detection

**Fig. 3:**
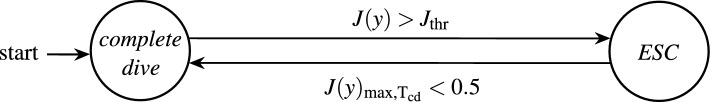
State machine describing the transitions between the two working modes *ESC* and *complete dive*.

Without loss of generality the thermocline is used as an example for an ocean layer. In the context of thermocline tracking the system $\Sigma (t,\varvec{u})$ refers to the AUV within the water body. The control input $\varvec{u}$ contains the position and pose of the AUV, which are realized by an underlying controller. Since ocean layers are either mainly horizontal or vertical the vector $\varvec{u}$ can be reduced to a scalar, in the case of the horizontal thermocline to the depth *z*, hence $\varvec{u} = z$. The system output $\varvec{y}$ are the measurements of the water properties, here the temperature *T*. The cost function is chosen as $J (\varvec{u}) = \frac{|\Delta T|}{|\Delta z|} = \frac{|T_i - T_j|}{|z_i - z_j|}$ where $i,j \in \mathbb {N}$ are discrete time indices with $i>j$. In the turning points of the AUV at the minimum and maximum of the perturbation signal this would lead to a division by very small numbers, in the worst case zero. To avoid this effect, the cost function is implemented as

6 $$\begin{aligned} J(\varvec{u}) = {\left\{ \begin{array}{ll} \frac{|\Delta T|}{|\Delta z|} \qquad & \text {if } \Delta z > z_\text {min} \\ 0 & \text {else} \end{array}\right. }, \end{aligned}$$

where $z_\text {min}$ is the adjustable minimum depth change. All ESC implementations have in common that a gradient of the cost function with respect to the system input has to be present, which cannot always be guaranteed^14^. Therefore the state machine shown in Fig. 3 is implemented with the UKF based ESC as the main working mode.

The AUV starts in the state *complete dive* in which it performs a complete downcast dive that is used for normalization of the cost function. The state changes to *ESC* when $J(\varvec{y}) > J_\text {thr}$ where $J_\text {thr} \in [0,1]$ due to the normalization. As a result, the approach does not need to be tuned specifically for the situation. Practical values for $J_\text {thr}$ are in the range between 0.2 and 0.8. The AUV only changes its working mode back to *complete dive* when $J(\varvec{y})_{{\text {max,T}}_\text {cd}} < 0.5$. Here $J(\varvec{y})_{{\text {max,T}}_\text {cd}}$ is the maximum cost function within the last $T_\text {cd} \,{\textrm{s}}$ with $T_\text {cd} \gg T_\text {p}$ and $T_\text {p}$ denoting the period length of the perturbation signal. Such a situation can occur if there is a large or sudden change of the environmental conditions or when the ESC drove the AUV into a local maximum/minimum of the cost function. If input constraints are known a priori, e.g. depth, velocity or acceleration limits, these could be integrated by means of suitable penalty functions, as illustrated in Fig. 2.

### AUV dynamics

**Fig. 4:**
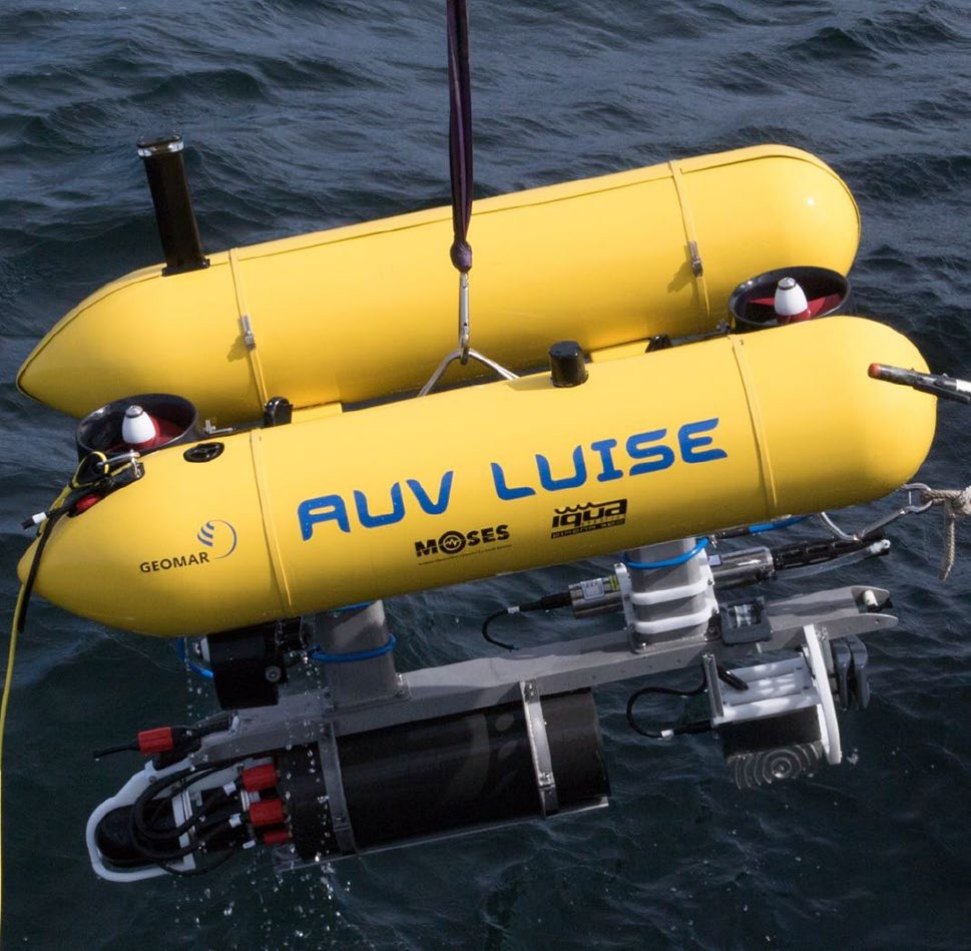
Girona500 AUV from IQUA robotics.

The AUV considered in this paper is the Girona500 AUV from IQUA Robotics shown in Fig. 4. It measures approximately $1 \; {\textrm{m}}$ x $1 \; {\textrm{m}}$ x $1.5 \; {\textrm{m}}$ (H x W x L), weighs between 140 and 200 kg, depending on the configuration and can operate in depths of up to $500 \; {\textrm{m}}$. The AUV has a 35-liter payload area for mission-specific instruments and can fly at speeds of up to 2 knots for 6 to 8 h. It is a hovering capable AUV that is equipped with five thrusters, two located at the top controlling heave and pitch, two at the back controlling thrust and yaw and the last one in the center of the AUV controlling the sway. Due to this configuration the AUV is underactuated and cannot control the roll motion but it is constructed such that the roll mode is stable. The mathematical model of the AUV dynamics used in this paper reads

7a $$\begin{aligned} \dot{\varvec{\eta }} =&\ R_{\varvec{\Theta }}(\varvec{\eta }) \varvec{\nu } \end{aligned}$$

7b $$\begin{aligned} M \dot{\varvec{\nu }} =&\ -C(\varvec{\nu })\varvec{\nu }-D(\varvec{\nu })\varvec{\nu } + B\varvec{\tau }, \end{aligned}$$

where $\varvec{\eta } = [x, y, z, \phi , \theta , \psi ]^\text {T}$ denotes the earth fixed position vector^15^. The coordinates *x*, *y*, *z* are defined in the North-East-Down (NED) frame and $\phi , \theta , \psi$ are the respective roll, pitch and yaw angles. The body fixed velocities surge, sway, heave, roll, pitch, and yaw yield the vector $\varvec{\nu } = [u,v,w,p,q,r]^\text {T}$ and $\varvec{\tau }=[\tau _u,\tau _v,\tau _w, \tau _q, \tau _r]^\text {T}$ is the control vector with the forces $\tau _u,\tau _v, \tau _w$ and moments $\tau _q,\tau _r$, respectively. The input matrix $B\in \mathbb {R}^{6\times 5}$ reads as

8 $$\begin{aligned} B = \begin{bmatrix}1 \;\, 0 \;\, 0 \;\, 0 \;\, 0 \\ 0 \;\, 1 \;\, 0 \;\, 0 \;\, 0 \\ 0 \;\, 0 \;\, 1 \;\, 0 \;\, 0 \\ 0 \;\, 0 \;\, 0 \;\, 0 \;\, 0 \\ 0 \;\, 0 \;\, 0 \;\, 1 \;\, 0 \\ 0 \;\, 0 \;\, 0 \;\, 0 \;\, 1\end{bmatrix}. \end{aligned}$$

Equation (7b) describes the AUV dynamics in body fixed coordinates with the inertia matrix *M*, Coriolis matrix *C* and damping matrix *D*. The transformation of the body fixed into earth fixed velocities is described in (7a). The acquisition of accurate modeling parameters is a very challenging and time consuming task since these models typically contain nonlinearities. Therefore *M* and *D* are estimated as diagonal matrices based on the specifications by IQUA Robotics. The underlying motion control system is chosen as a PID controller for each controllable degree of freedom. A thruster allocation matrix is used to convert the desired force of each degree of freedom into the required force of each thruster.

**Fig. 5:**
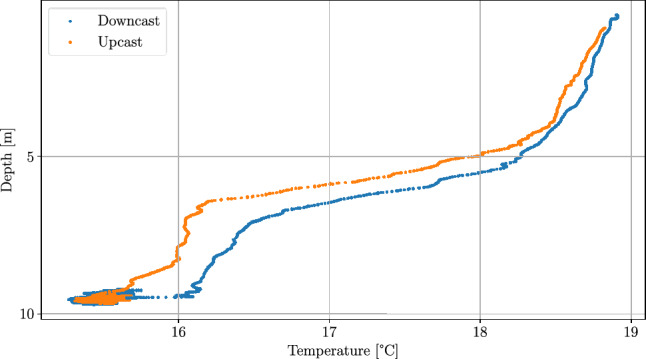
Scatter plot of the AUV depth against temperature for an AUV survey mission in the Baltic Sea, August 2020.

### Simulation framework

For the simulations a framework is setup, which is based on the Robot Operating System (ROS), Gazebo and the Unmanned Underwater Vehicles (UUV) simulator^16–18^. ROS provides fundamental functionalities such as standardized communication among software entities and coordinate transformations and can therefore be seen as the middleware for the other two. Gazebo is a robot simulator that promises realistic simulation results due to the build in Dynamic Animation and Robotics Toolkit (DART) physics engine. Since Gazebo is built for land-based robots the UUV simulator is used which integrates the hydrodynamic forces and moments as custom plugins in the physics engine.

### Simulation data

In Fig. 5 an example of CTD data is shown as scatter plot of the AUV depth against temperature for a classical lawnmower survey mission with the GIRONA500 AUV. It can be seen that there is a distinct difference in the measurement between the downcast and upcast, which looks similar to a hysteresis. This could theoretically be caused by internal waves but since this effect can be observed in all our AUV missions with CTD measurements it has two possible reasons: First the used Seabird scientific sbe49FastCat has a characteristic sensor response time. Second and probably equally relevant is that while the AUV is driving downwards the CTD measures the undisturbed water, which is in contrast to driving upward, where bottom water is pushed upwards by the two hulls at the top of the AUV leading to turbulent mixing of the water. This has the severe consequence that a single profile cannot be used to simulate realistic temperature measurements of the GIRONA500 AUV.

### Emulation of the sensor effects

**Fig. 6:**
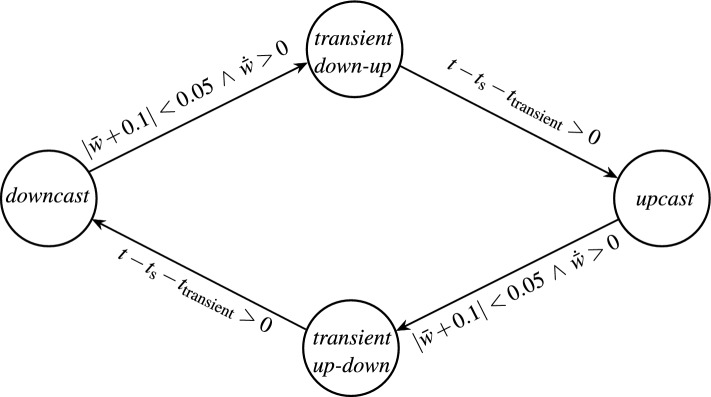
State machine describing the transitions between the down- and upcast.

To emulate the effects of the CTD sensor an upcast and a downcast profile are used which are linearly interpolated at the turning points of the vertical AUV motion. This is realized by the state machine in Fig. 6. Here *t* is time, $t_\text {s}$ denotes the start time of the transient/interpolation, and *w* is the heave velocity of the AUV. Furthermore $|\cdot |$ denotes the absolute and $\bar{\cdot }$ the moving average with length $t_\text {win}$ of a variable. In the states *downcast* and *upcast* the temperature measurements are only depending on a single profile. The condition for a state transition from *downcast* to *upcast* via *transient down-up* was chosen as $|\bar{w} + 0.1| < 0.05$ and $\dot{\bar{w}} > 0$. This describes the situation, where the AUV is about to change its vertical driving direction. The condition for a state transition from *upcast* to *downcast* follows analogously. The interpolation is performed in the states *transient down-up* and *transient up-down* and takes the time $t_\text {transient}$. The conditions $t-t_\text {s} - t_\text {transient} > 0$ and $t-t_\text {s} - t_\text {transient} < 0$ for leaving the *transient down-up* and *transient up-down* state, respectively, ensure that the interpolation is performed completely to prevent the system from oscillating. If desired, an input constraint could be implemented in the UKF based ESC for the time of the interpolation by means of a penalty function, as described in the next two sections, to prevent unforeseen behavior. The simulated temperature sensor measurement *T*(*z*) is computed as

9 $$\begin{aligned} T(z) ={\left\{ {\left\{ \begin{array}{ll} T_\text {dc}(z) + \xi \hspace{6cm} & downcast \\ T_\text {uc}(z) + \xi & upcast \\ \frac{t-t_\text {s}}{t_\text {transient}} \cdot T_\text {dc}(z) + \left( 1-\frac{t-t_\text {s}}{t_\text {transient}}\right) \cdot T_\text {uc}(z) + \xi & \textit{transient up-down} \\ \left( 1-\frac{t-t_\text {s}}{t_\text {transient}}\right) \cdot T_\text {dc}(z) + \frac{t-t_\text {s}}{t_\text {transient}} \cdot T_\text {uc}(z) + \xi & \textit{transient down-up} \end{array}\right. }\right. }, \end{aligned}$$

where *z* is the depth, $T_\text {dc}(z)$ and $T_\text {uc}(z)$ are the original measurement data for downcast and upcast, respectively, and $\xi$ is white measurement noise.

## Results

### Simulation results

As pointed out in the introduction, the data in von See et al. (2021) did not consider the effect described in the Simulation data section. In this paper the performance of the UKF based ESC in the case of complex ocean layers and realistic sensor dynamics is investigated. For the simulation a CTD profile measured with a ship based CTD was used because a similarly complex profile was not yet measured with our GIRONA500 AUVs. Since the sensor response of our ship based CTD during upcast is different to the response of our AUV’s CTD the effects in the upcast data were emulated in the simulation. The upcast CTD data was shifted $1 \, {\textrm{m}}$ towards the surface and to compensate the shift in the upper and lower regions of the water column the data was saturated close to the surface and expanded close to the sea floor.

**Fig. 7:**
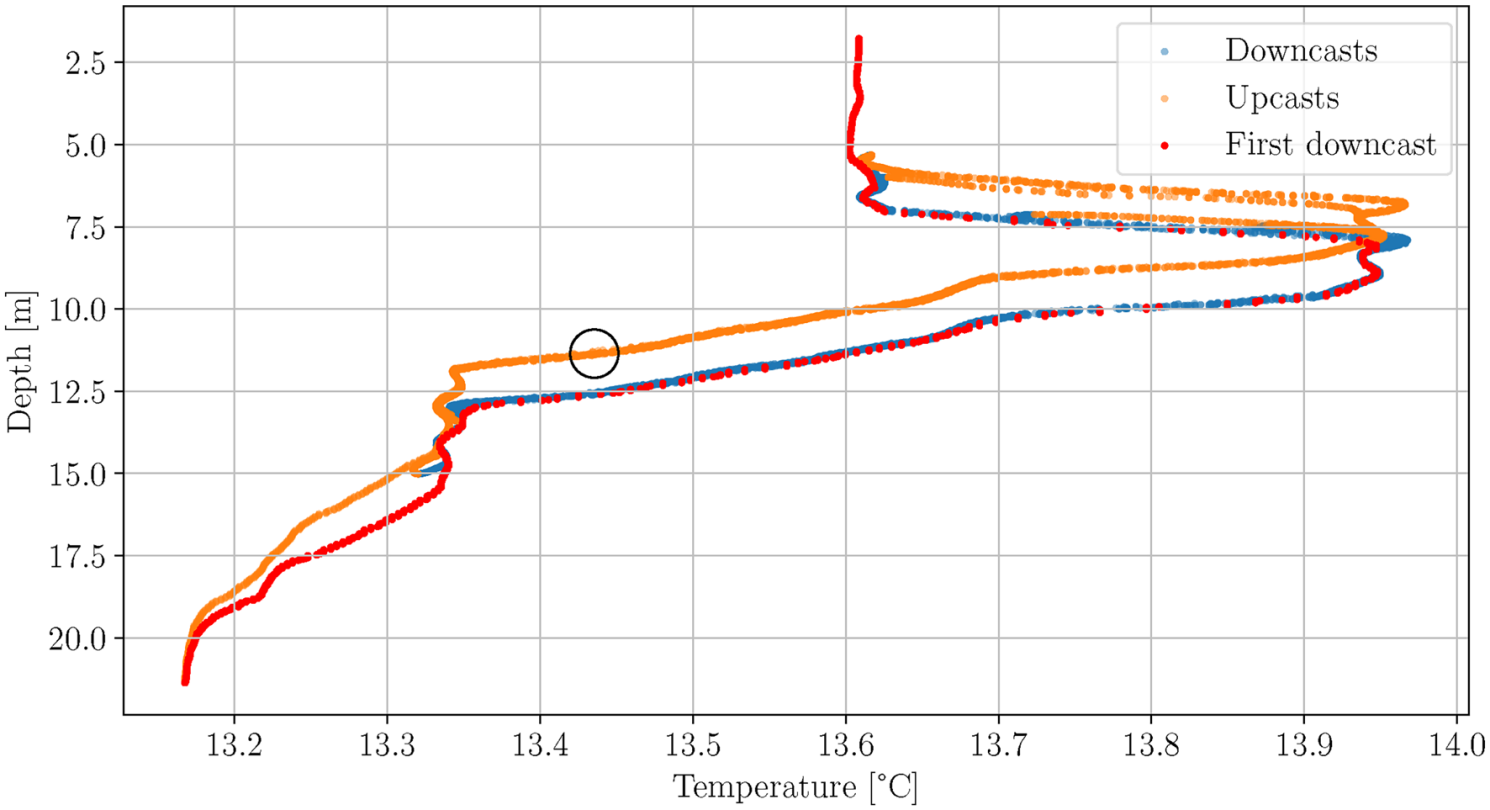
Scatter plot of the AUV depth against temperature for the UKF based ESC thermocline tracking simulation. The black circle marks the point at which the ESC is started.

**Fig. 8:**
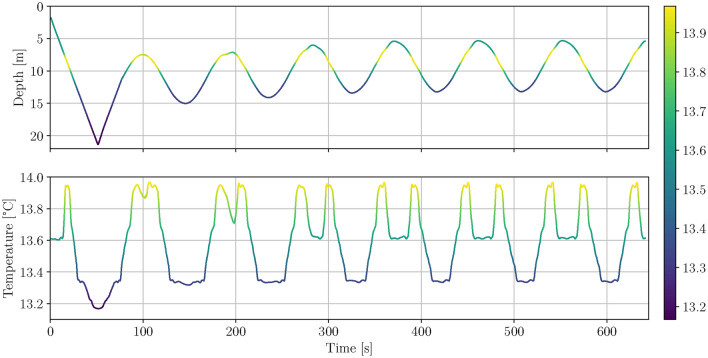
Depth and temperature plotted over time for the UKF based ESC thermocline tracking simulation. Both plots are color-coded based on the temperature data.

In Fig. 7 the AUV depth against temperature for the UKF based ESC thermocline tracking simulation is shown as a scatter plot. The red dots indicate the first downcast, orange dots the upcasts and blue dots the subsequent downcasts. The starting point of the UKF based ESC is marked by the black circle. It can be seen that there are two ocean layers, one from approx. $7 \text { to }8 \, {\textrm{m}}$ and the other from approx. $9.5 \text { to }13 \, {\textrm{m}}$. The water between $15.5 \text { and } 20 \, {\textrm{m}}$ is not considered an ocean layer because the gradient of temperature with respect to depth is much smaller than in the two other regions. The ESC perturbation amplitude and frequency are chosen such that the resulting amplitude covers approx $7.5\,{\textrm{m}}$, thus $1.5 {\textrm{m}}$ more than the thickness of the warm water intrusion that is marked by the two thermoclines. In Fig. 8 the depth of the AUV resulting from the UKF based ESC and the corresponding temperature are plotted over time. In the plot at the top it can be seen that the AUV first performs the initial dive from $0 \text { to approx. } 50 \, {\textrm{s}}$ followed by the upcast until $77 \, {\textrm{s}}$ and corresponding depth of $11.3 \, {\textrm{m}}$. At this point the ESC is started, which fine tunes the depth within three perturbation periods such that the range from approx. $5.5 \text { to } 13\,{\textrm{m}}$ is covered. During the first perturbation period only a small part of the upper layer is covered but from approx. $280 \, {\textrm{s}}$ onward also the complete upper layer is tracked. This shows that the UKF based ESC is able to track more than one ocean layer even in the presence of sensor delay and hysteresis effects. The parameters used in the simulation are listed in Table 1.

**Table 1:** ESC parameters used in the simulation.

Simulation data parameters	
Moving average window length $t_\text {win}$	$1 \, {\textrm{s}}$
Transient time $t_\text {transient}$	$15 \, {\textrm{s}}$

ESC parameters	
Period perturbation signal $T_\text {p}$	$91\, {\textrm{s}}$
Amplitude perturbation signal $A_\text {p}$	$0.0135 \, {\textrm{m}}$
Signal shape perturbation signal	cosine
Optimization gain *k*	$1.5 \cdot 10^{-4}$
Covariance *R*	$3 \cdot 10^{-5}$
Covariance *Q*	$3 \cdot 10^{-5}$
Parameter UKF $\alpha$	1
Parameters UKF $\beta , \gamma$	0
Minimum depth change $z_\text {min}$	$0.05\, {\textrm{m}}$
AUV surge speed *u*	$0.4 \frac{{\textrm{m}}}{{\textrm{s}}}$
Moving average window length $t_\text {win}$	$2\, {\textrm{s}}$
Cost function threshold $J_\text {thr}$	0.25

### Results from AUV dives in the Baltic Sea

On 28th October 2022 the UKF based ESC was used to investigate the water layering in the Lübeck Bay in the Baltic Sea. The amplitude of the perturbation signal was chosen such that the AUV covers $\approx$ 10 m.

**Fig. 9:**
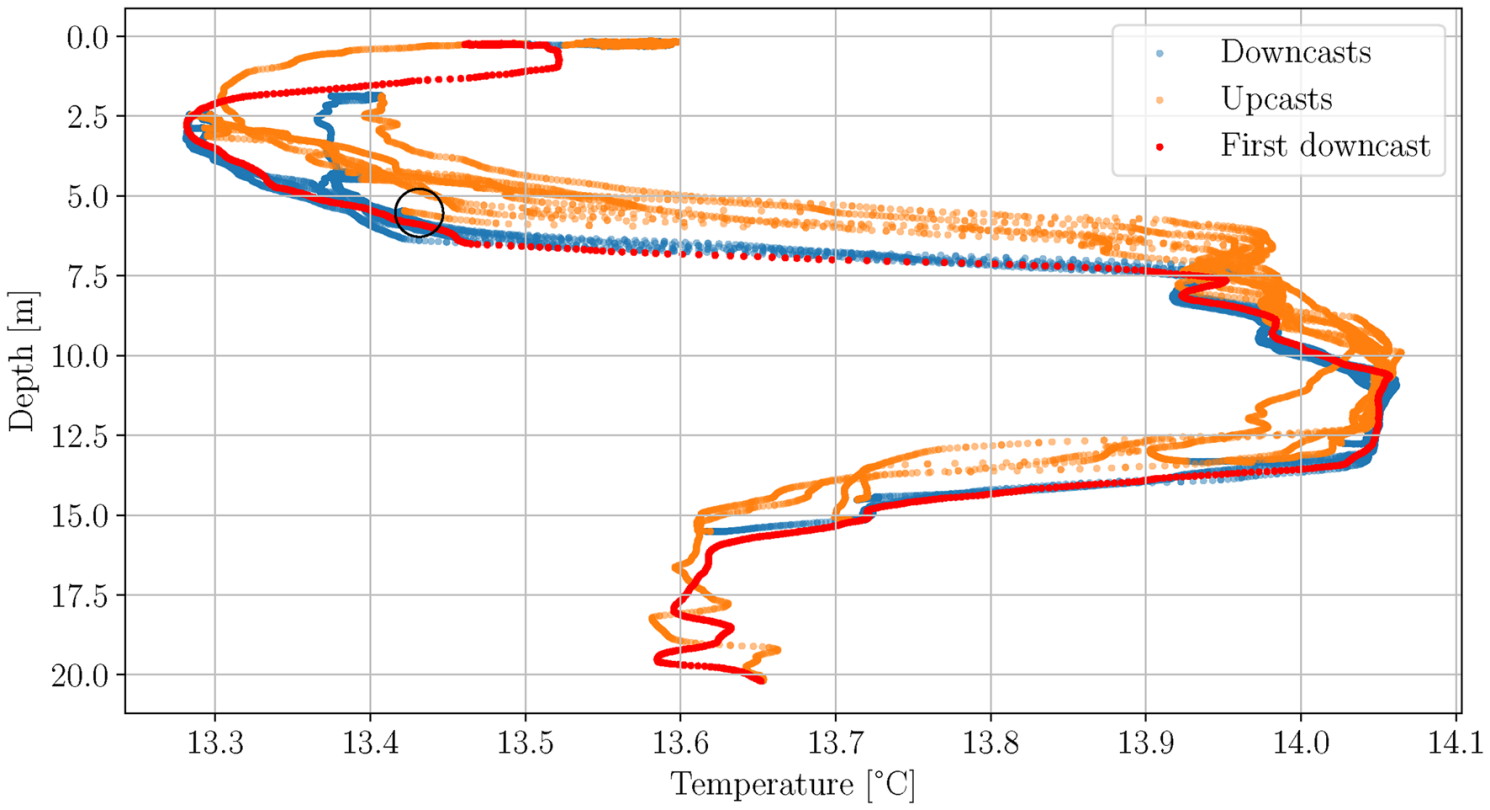
Scatter plot of the AUV depth against temperature for the UKF based ESC thermocline tracking dives in the Baltic Sea, October 2022. The black circle marks the point at which the ESC is started.

**Fig. 10:**
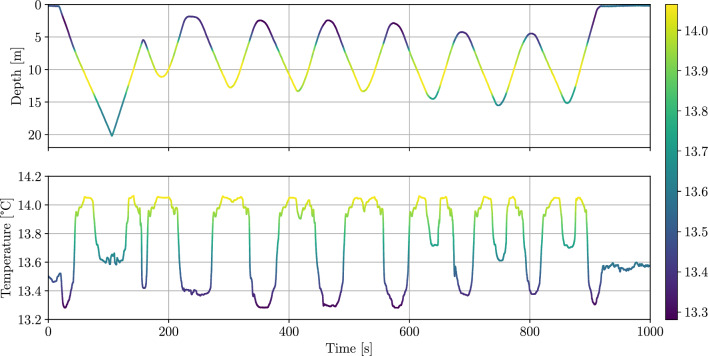
Depth and temperature plotted over time for the UKF based ESC thermocline tracking dives in the Baltic Sea, October 2022. Both plots are color-coded based on the temperature data.

Figure 9 shows the depth-temperature profile as scatter plot. The red dots indicate the first downcast, which can be seen as the reference for the dive, blue dots represent the measurements during downcasts and the orange dots measurements during upcasts. The starting point of the UKF based ESC is marked by the black circle. It can be seen that there are two main ocean layers, the upper one from approximately 3 to 7.5 ${\textrm{m}}$, where the largest gradient is from 6.5 to 7.5 ${\textrm{m}}$, and the lower one from 13.5 to 15.5 ${\textrm{m}}$. The decrease in temperature close to the surface is scientifically not interesting as this is caused by solar radiation making it an unstable ocean layer that has no persistent effect on the chemical or biological composition of the ocean. The differences of the measurements during upcast and downcast described in the simulation data section are also visible here. Additionally it can be observed that the measurement uncertainty for the temperature is larger during upcasts than during downcasts. This is also due to the mixing of the water by the two hulls at the top of the AUV. Figure 10 shows the depth and temperature measurements over time. An initial complete downcast can be seen from $t\approx 0$ to $100 \, {\textrm{s}}$. The UKF based ESC is started at $t \approx 175 \, {\textrm{s}}$ in $5.5 \, {\textrm{m}}$ depth and within the first perturbation period the range from 1.8 to $11 \,{\textrm{m}}$ is covered, which represents the upper ocean layer but no part of the lower layer. It can be seen that the ESC drives the AUV to greater depth so that at $t \approx 630 \, {\textrm{s}}$ also the lower layer is tracked partly and at $t \approx 750 \, {\textrm{s}}$ completely. This comes at the cost that the upper layer is not covered completely anymore but only the part with the highest gradient. By construction the ESC drives the AUV to the working point corresponding to the largest cost function, which is in this case a trade-off between the two layers. The ESC parameters that were used during the AUV dives are listed in Table 2.

**Table 2:** ESC parameters used in the filed test in the Baltic Sea.

Period perturbation signal $T_\text {p}$	$111\, {\textrm{s}}$
Amplitude perturbation signal $A_\text {p}$	$0.032 \, {\textrm{m}}$
Signal shape perturbation signal	cosine
Optimization gain *k*	$8 \cdot 10^{-4}$
Covariance *R*	$5 \cdot 10^{-6}$
Covariance *Q*	$5 \cdot 10^{-5}$
Parameter UKF $\alpha$	1
Parameters UKF $\beta , \gamma$	0
Minimum depth change $z_\text {min}$	$0.2\, {\textrm{m}}$
AUV surge speed *u*	$0.4 \frac{{\textrm{m}}}{{\textrm{s}}}$
Moving average window length $t_\text {win}$	$2\, {\textrm{s}}$
Cost function threshold $J_\text {thr}$	0.6

## Discussion

Signal based optimization techniques such as the proposed UKF based ESC face two challenges. First, a gradient of the cost function has to be present in the working point of the system and second, prior knowledge of the expected dimension of the phenomenon is required. The first is addressed by introducing a two stage approach ensuring that the ESC is only started in regions with significant gradient information. The second is less severe in the marine context as good estimates of the thickness of most ocean layers can be made. If desired an extension can be added to ensure complete sampling of the layer. This implies a trade-off, which bears the risk of either not sampling the whole marine water layer or increasing the mission time compared to the ideal case and therefore degrading the efficiency. This trade-off applies not only to ESC but also to threshold-based methods, where a suboptimal choice of the threshold, which defines the ocean layer, can lead to the same effects. Therefore an extension depth is also used in Zhang et al. (2012)^8^ and Feng et al. (2021)^9^. In the case of thin ocean layers, e.g. in coastal regions as shown in the results above, extending the estimated layer depth does not result in much longer mission times compared to the overall mission time and is therefore feasible. In the case of large ocean layers, e.g. upwelling fronts, a possible improvement of the presented method could be to implement an automatic tuning of the ESC parameters based on the measured data. The tuning is mainly aimed at the frequency and amplitude of the perturbation signal. By this, the influence of prior knowledge on the effectiveness of the approach could be reduced. Despite the limitations of the proposed method, the data from the simulation and field test show that the proposed UKF based ESC is able to detect and track complex ocean layers subject to sensor delays and hysteresis.

All papers mentioned in the introduction except Feng et al. (2021) present very smooth data similar to the thermocline shown in Fig. 5 but non-ideal ocean layers, as the one shown in Fig. 9, are typically found in nature, particularly in shallower water and when the water layering is changed e.g. during seasonal changes. Compared to the methods proposed in these papers the presented approach takes more time until the target layer is completely sampled. The duration depends on the complexity of the ocean layer and is in the range of $2\, T_p$ to $4 \, T_p$, where $T_p$ is the period length of the perturbation signal. The advantage on the other hand is that the sampling is more even, meaning that the depth range that the AUV samples is constant making it more robust against small scale fluctuations of the ocean layer and sensor delays. This is advantageous because e.g. in Zhang et al. (2012) the sampled depth per ascent or descent leg is variable. For a presented field test the sampled depth range varies from approx. 5 to 9 meters, as a result the ocean layer is not covered completely all the time. Additionally, the UKF based ESC can easily be tuned for situations with complex water layering, where pure threshold-based algorithms reach their limits as pointed out in Feng et al. (2021)^9^. The method proposed by Feng et al. (2021) is also able to find and track complex ocean layers. For environmental situations as shown in Fig. 9 both, the method from Feng et al. (2021) and the method presented in this paper, can lead to the same sampling range. Due to the different techniques used in these two approaches they are very different in the way they have to be tuned. For the approach in Feng et al. (2021) the temperature gradient that defines the thermocline has to be estimated apriori whereas in the approach presented in this paper the thickness of the thermocline has to be estimated apriori. Both quantities fluctuate seasonally within the year and it depends e.g. on the region, water depth and morphology which quantity changes more. A scenario where the approach presented in this paper is beneficial is when there are two ocean layers present, one near the surface and another near the seafloor. This can for example happen in the Baltic Sea when there is a salt water intrusion from the North Sea. The saltier and therefore denser water from the North Sea does not mix with the water from the Baltic Sea but spreads out on the sea floor, filling up deeper basins, an important mechanism to oxygenate the Baltic Sea. This results in a boundary layer between the brackish water of the Baltic Sea and the salt water near the sea floor in addition to the thermocline near the surface. In such a situation the method from Feng et al. (2021) would sample nearly the whole water column. The approach presented in this paper could be tuned to also sample nearly the whole water column but can additionally be tuned to sample one of the layers individually which gives the AUV operator more flexibility. Especially if only one of these layers is of interest, the AUV mission time can be significantly reduced.

## Supplementary Information

Supplementary Information 1.

Supplementary Information 2.

## Data Availability

The authors declare that the data supporting the findings of this study are available within the paper and its supplementary information files.
