# Supplementary material for: Detection and tracking of ocean layers using an AUV with UKF based extremum seeking control in the Baltic Sea
Source: Sci Rep. 2024 Sep 4;14:20629. doi: 10.1038/s41598-024-70775-y (PMC11374998; doi:10.1038/s41598-024-70775-y)
Supplement: Supplementary file 1 — Supplementary Information 1. [file 41598_2024_70775_MOESM1_ESM.pdf]

Summary of the Unscented Kalman Filter

The UKF is an extension of the Kalman Filter, which is suited for state estimation of nonlinear processes. In contrast to the widely used Extended Kalman Filter (EKF) it does not use a linearization of the nonlinear function but instead uses the unscented transform (UT) to capture the nonlinearities. Consider the problem

$$\begin{aligned} x_{k+1} &= f(x_k, u_k) + w_k \\ y_k &= g(x_k) + l_k \end{aligned} \tag{1}$$

where x_k is a state vector at the discrete time k with mean $\bar{x}_k$ and covariance P_{x_k}, u_k is the system input, $f(x_k, u_k)$ and $g(x_k)$ are nonlinear functions, and w_k and l_k are the process and measurement noise with covariance R_k^w and R_k^l, respectively. The basic idea of the UT is to choose a set of sigma points $\mathscr{X}_{i,k}$ and corresponding weights W_i, where $i \in [0, 2n+1]$ and n is the number of dimensions of x. These deterministically chosen points are propagated through the nonlinear function $f(x,u)$ and the statistics are calculated from the transformed points. The sigma points and their weights have to satisfy certain conditions, see e.g. Julier and Uhlmann (2004) for details[1]. A classical choice for this is

$$\begin{aligned} \mathscr{X}_{0,k-1} &= \bar{x}_{k-1} \\ \mathscr{X}_{i,k-1} &= \bar{x}_{k-1} + \left(\sqrt{(n+\lambda)P_{x_{k-1}}}\right)_i & i &= 1, ..., n \\ \mathscr{X}_{i,k-1} &= \bar{x}_{k-1} - \left(\sqrt{(n+\lambda)P_{x_{k-1}}}\right)_{i-n} & i &= n+1, ..., 2n \\ W_0^{(m)} &= \frac{\lambda}{n+\lambda} \\ W_0^{(c)} &= \frac{\lambda}{n+\lambda} + (1-\alpha^2+\beta) \\ W_i^{(m)} &= W_i^{(c)} = \frac{1}{2(n+\lambda)} & i &= 1, ..., 2n \end{aligned} \tag{2}$$

where $\left(\sqrt{(n+\lambda)P_{x_{k-1}}}\right)_i$ is the i-th row of the matrix square root and $\lambda = \alpha^2(n+\kappa) - n$[1,2]. The parameters α, β and κ are tuning parameters and $W_0^{(m)}$ and $W_0^{(c)}$ are the first and the second order weights, respectively. The time update of the filter is performed as

$$\mathscr{X}_{k|k-1} = f(\mathscr{X}_{k-1}, u_{k-1}) \tag{3}$$

$$\bar{x}_k^- = \sum_{i=0}^{2n} W_i^{(m)} \mathscr{X}_{i,k|k-1} \tag{4}$$

$$P_{x_k}^- = \sum_{i=0}^{2n} W_i^{(c)} (\mathscr{X}_{i,k|k-1} - \bar{x}_k)(\mathscr{X}_{i,k|k-1} - \bar{x}_k)^{\mathrm{T}} + R^w \tag{5}$$

$$\mathscr{Y}_{k|k-1} = g(\mathscr{Y}_{k-1}) \tag{6}$$

$$\bar{y}_k^- = \sum_{i=0}^{2n} W_i^{(m)} \mathscr{Y}_{i,k|k-1}. \tag{7}$$

Here $\mathscr{X}_{k-1}$ is the matrix of the $2n+1$ sigma points $\mathscr{X}_{i,k-1}$ and $f(\mathscr{X}_{k-1}, u_{k-1})$ denotes the column-wise application of $f(x,u)$. This applies analogously to (6). The measurement update is given by

$$P_{y_k y_k} = \sum_{i=0}^{2n} W_i^{(c)} (\mathscr{Y}_{i,k|k-1} - \bar{y}_k^-)(\mathscr{Y}_{i,k|k-1} - \bar{y}_k^-)^{\mathrm{T}} + R^l \tag{8}$$

$$P_{x_k y_k} = \sum_{i=0}^{2n} W_i^{(c)} (\mathscr{X}_{i,k|k-1} - \bar{x}_k^-)(\mathscr{Y}_{i,k|k-1} - \bar{y}_k^-)^{\mathrm{T}} \tag{9}$$

$$\mathscr{K}_k = P_{x_k y_k} P_{y_k y_k}^{-1} \tag{10}$$

$$x_k = \bar{x}_k^- + \mathscr{K}_k (y_k - \bar{y}_k^-) \tag{11}$$

$$P_{x_k} = P_{x_k}^- - \mathscr{K}_k P_{y_k y_k} \mathscr{K}_k^{\mathrm{T}}. \tag{12}$$

Even though the approach of the UT bears some similarities to particle filters, there are some distinct differences. The most important ones are that the order of samples needed is much lower for the UT and that these samples are not chosen randomly but deterministically so that they e.g. have a given mean and covariance[1]. Furthermore the approximation of the statistics with the UT are accurate to the third order for Gaussian inputs and accurate to at least the second order for inputs with other distributions whereas the approximations with the EKF are only accurate to the first order[3]. In addition the UKF does not require the calculation of a Jacobian matrix, which does not always exist and even if it does its calculation is difficult and error prone[1]. The computational complexity of the standard UKF is $\mathcal{O}(n^3)$ but the implementation as square-root UKF has complexity $\mathcal{O}(n^2)$, which is the same as for the EKF[2]. For a more detailed analysis of the UKF the interested reader is referred to Wan and Van Der Merwe (2000), Van Der Merwe and Wan (2001) and Julier and Uhlmann (2004)[1–3].

References

1. Julier, S. & Uhlmann, J. Unscented filtering and nonlinear estimation. *Proc. IEEE* **92**, 401–422 (2004).

2. Van Der Merwe, R. & Wan, E. A. The square-root unscented kalman filter for state and parameter-estimation. In *2001 IEEE international conference on acoustics, speech, and signal processing. Proceedings (Cat. No. 01CH37221)*, vol. 6, 3461–3464 (IEEE, 2001).

3. Wan, E. A. & Van Der Merwe, R. The unscented kalman filter for nonlinear estimation. In *Proceedings of the IEEE 2000 Adaptive Systems for Signal Processing, Communications, and Control Symposium (Cat. No. 00EX373)*, 153–158 (Ieee, 2000).
